# Interest of the Robotic Approach for Pancreaticoduodenectomy in Elderly Patients in a Setting of Limited Robotic Platform Access: A Propensity Score-Matched Comparison with Open Surgery

**DOI:** 10.3390/jcm15041520

**Published:** 2026-02-14

**Authors:** Edouard Wasielewski, Antoine Castel, Hector Prudhomme, Kevin Preault, Salaheddine Abdennebi, Marie Livin, Aude Merdrignac, Fabien Robin, Laurent Sulpice

**Affiliations:** 1 Department of Hepatobiliary and Digestive Surgery, University Hospital Center, Rennes 1 University, 35000 Rennes, France; edouard.wasielewski@chu-rennes.fr (E.W.); antoine.castel@chu-rennes.fr (A.C.); hector.prudhomme@chu-rennes.fr (H.P.); kevin.preault@chu-rennes.fr (K.P.); salaheddine.abdennebi@chu-rennes.fr (S.A.); marie.livin@chu-rennes.fr (M.L.);; 2 Hepatobiliary Surgery and Liver Transplantation Research Unit, University Hospital Center, INSERM, CIC1414, 35000 Rennes, France; 3 OSS (Oncogenesis Stress Signaling) Laboratory, INSERM, University of Rennes, UMR_S 1242, Centre de Lutte Contre le Cancer Eugène Marquis, 35042 Rennes, France

**Keywords:** minimally invasive surgery, robotic-assisted surgery, robotic pancreaticoduodenectomy, elderly patients, surgical outcomes

## Abstract

**Background**: With population aging and the increasing incidence of pancreatic and periampullary malignancies, more elderly patients are being considered for pancreaticoduodenectomy (PD). Although robotic pancreaticoduodenectomy (RPD) is steadily adopted, evidence regarding its safety in patients aged ≥ 75 years remains limited, particularly in centers with restricted access to robotic platforms. **Materials and Methods**: We conducted a retrospective single-center study including patients who underwent PD between January 2019 and September 2025. Outcomes after RPD were compared between patients aged < 75 and ≥75 years. In addition, elderly patients undergoing RPD were compared with elderly patients undergoing open pancreaticoduodenectomy (OPD) using 1:2 propensity score matching. The primary endpoint was major postoperative morbidity (Clavien–Dindo grade ≥ III). **Results**: Among 525 PDs, 130 (25%) were performed robotically, including 29 patients aged ≥ 75 years. Within the RPD cohort, age ≥ 75 years was not associated with an increased risk of major complications compared with younger patients (OR 0.68, 95% CI 0.23–1.76; *p* = 0.45), nor with higher 90-day mortality. In the propensity score-matched elderly cohort, major morbidity was similar between RPD and OPD (10% vs. 7%; *p* = 0.68). RPD was associated with a significantly lower 30-day readmission rate, despite a higher incidence of delayed gastric emptying, mainly driven by mild (grade A) cases. **Conclusions**: RPD appears to be safe in carefully selected patients aged ≥ 75 years, with morbidity and mortality comparable to those observed in younger RPD patients and in elderly patients undergoing open surgery. These findings support the selective use of RPD in elderly patients, even in centers with limited access to robotic platforms.

## 1. Introduction

With increasing life expectancy, population aging has become a major global public health challenge. According to the United Nations, the proportion of individuals aged over 65 years is expected to double between 2024 and 2074, while the number of adults aged 80 years and older is projected to triple. This demographic group is characterized by a high prevalence of comorbidities, which is associated with an increased risk of perioperative complications. In this context, there is a critical need to better characterize surgical risks in elderly patients and to identify strategies aimed at minimizing perioperative morbidity.

At the same time, the incidence of pancreatic and periampullary malignancies increases with age. As a result, a growing number of elderly patients are being considered for pancreaticoduodenectomy (PD), which remains the only potentially curative treatment [1,2]. In patients aged ≥ 75 years, surgical decision-making must therefore rely on a rigorous benefit–risk assessment, balancing expected gains in survival and quality of life against the risks of postoperative mortality and severe complications.

Minimally invasive approaches have become steadily adopted in gastrointestinal surgery. However, their application in PD remains controversial, given the technical complexity of the procedure [3,4]. Nevertheless, growing evidence suggests potential advantages over the open approach, particularly with robotic pancreaticoduodenectomy (RPD), whose adoption has expanded as it addresses several technical limitations of laparoscopy [5]. Comparative studies have reported shorter hospital stays and reduced postoperative morbidity compared with open pancreaticoduodenectomy (OPD). However, most available data originate from high-volume centers with extensive access to robotic platforms, and evidence in elderly and very elderly populations remains limited [6,7]. Recently, Castel et al. demonstrated that limited access to robotic platforms does not preclude the safe implementation of RPD, with outcomes comparable to those of open surgery even in resource-constrained settings [8]. The applicability of these findings to elderly patients, however, has yet to be fully established.

The aim of this study was to evaluate the perioperative safety and feasibility of RPD in patients aged ≥ 75 years, and to compare outcomes with both younger patients and elderly patients undergoing OPD.

## 2. Materials and Methods

### 2.1. Study Design

We conducted a retrospective, single-center study based on a prospectively maintained database including all patients who underwent PD between 1 January 2019 and 30 September 2025, at a high-volume pancreatic surgery center in France. All RPD and more than 90% of OPD were performed by a single surgeon with formal training in robotic surgery. All consecutive patients undergoing PD during the study period for either benign or malignant disease were included. For malignant tumors, surgical indications were systematically reviewed and validated by a multidisciplinary tumor board. All procedures were performed with curative intent. Indications for surgery included pancreatic and periampullary lesions, such as pancreatic ductal adenocarcinoma, acinar cell carcinoma, duodenal adenocarcinoma, ampullary tumors (benign and malignant), intraductal papillary mucinous neoplasms (IPMNs), neuroendocrine tumors, and cholangiocarcinoma. Prior to analysis, all pancreaticoduodenectomies performed using a purely laparoscopic approach, as well as all total pancreatectomies (both laparoscopic and open), were excluded. For the purposes of this study, patients were classified into two groups: elderly and younger patients. In accordance with the literature, including the EPIDOS study, and the World Health Organization definition, elderly patients were defined as those aged ≥ 75 years at the time of surgery [9,10,11,12].

### 2.2. Ethics

The study was conducted in accordance with the ethical principles of the 2013 Declaration of Helsinki. The study protocol was approved by the Rennes University Hospital Ethics Committee (approval No. 25.20). The requirement for individual informed consent was waived due to the retrospective design of the study and the full anonymization of patient data.

### 2.3. Data Collection

Data were extracted from patients’ electronic medical records. Preoperative variables included age, sex, lifestyle factors (alcohol and tobacco use), body mass index (BMI), American Society of Anesthesiologists (ASA) score, and Charlson Comorbidity Index (CCI), comorbidities, presenting symptoms at diagnosis, any preoperative drainage procedures, and use of neoadjuvant therapy [13,14]. Intraoperative variables included operative time (minutes), pancreatic texture, and associated procedures such as venous resection. Postoperative outcomes included length of hospital stay, duration of intensive care unit (ICU) admission, all postoperative complications occurring during hospitalization, need for reintervention, blood transfusions, unplanned hospital readmission (defined as any unplanned rehospitalization within 30 days after surgery, whether at our institution or an external facility), and 30-and 90-day mortality.

### 2.4. Definitions

Postoperative complications were graded according to the Clavien–Dindo classification, with major morbidity defined as grade III or higher [15]. Postoperative pancreatic fistula (POPF), postpancreatectomy hemorrhage (PPH), and delayed gastric emptying (DGE) were defined and classified according to the criteria of the International Study Group of Pancreatic Surgery (ISGPS) [16,17,18].

### 2.5. Procedure and Perioperative Management

During the resection phase, OPD was systematically performed with pylorus resection. Digestive reconstruction was carried out according to the standard Child technique, including creation of a transmesocolic jejunal limb, an end-to-side pancreaticojejunostomy constructed with interrupted sutures, followed by an end-to-side hepaticojejunostomy using continuous running sutures, and a hand-sewn end-to-side or side-to-side gastrojejunostomy. The jejunal limb used for the gastrojejunostomy was positioned in an antemesocolic fashion.

For the robotic approach, all patients underwent pylorus-resecting pancreaticoduodenectomy with reconstruction based on the Child technique. The procedure followed the 17 standardized operative steps for RPD described by Giulianotti et al. [19]. After completion of the resection phase, the first jejunal loop was passed posterior to the superior mesenteric vessels and advanced into the supramesocolic compartment. Reconstruction was then performed sequentially with a pancreatojejunostomy, a hepaticojejunostomy, and finally a gastroenterostomy. During the early phase of the learning curve, the pancreatojejunostomy and hepaticojejunostomy were routinely constructed using interrupted sutures, with a gradual transition toward increased use of continuous running sutures over time. The gastroenterostomy was created using a stapled lateral side-to-side technique and was initially positioned in a supramesocolic but non-antecolic configuration. In all cases, intra-abdominal drains were systematically placed to enable measurement of drain amylase levels on postoperative days 3 and 5, facilitating early detection of postoperative pancreatic fistula. Drain catheters were positioned both anterior and posterior to the pancreatojejunostomy. In patients with severe malnutrition, a nasojejunal feeding tube was inserted intraoperatively. Prophylactic antibiotics were administered 30 min before skin incision. When an endoscopic biliary stent was present, postoperative antibiotic therapy was extended to five days and adapted according to routinely obtained intraoperative bile cultures, irrespective of stent presence. It should be noted that during the study period, access to the robotic surgical platform was limited to one to two sessions per month during the first three years, subsequently increasing to two to three sessions per month thereafter.

### 2.6. Statistical Analysis

Categorical variables are presented as absolute frequencies with corresponding percentages and were compared using the chi-square test or Fisher’s exact test, as appropriate. Continuous variables are reported as medians with interquartile ranges (IQR) and were compared using Student’s *t* test or the Mann–Whitney U test, as appropriate. To evaluate the safety of RPD compared with OPD, a 1:2 propensity score matching strategy was applied. Patients were matched on age, sex, Charlson Comorbidity Index (CCI), body mass index (BMI), and presumed diagnosis. Presumed diagnosis was used to reflect the preoperative clinical indication guiding surgical decision-making and to ensure applicability of the matching strategy across both benign and malignant conditions. Covariate balance after matching was assessed using standardized mean differences (SMD), with values below 0.10 considered indicative of adequate balance. In the propensity score-matched cohort (2:1), a logistic regression model with robust standard errors clustered by matched sets was used to account for within-pair correlation. The primary endpoint was major postoperative complications, defined as Clavien–Dindo grade III or higher. The objective of this study was intentionally limited to the assessment of perioperative safety and feasibility, defined by postoperative morbidity and mortality. Analyses did not include evaluation of oncological outcomes, functional recovery, or cost-effectiveness. Secondary endpoints included length of hospital stay, need for surgical reintervention, hospital readmission rate, incidence of specific postoperative complications, and 30- and 90-day postoperative mortality. All statistical analyses were performed using R statistical software (version 4.3.3; R Foundation for Statistical Computing, Vienna, Austria).

## 3. Results

**Figure 1:**
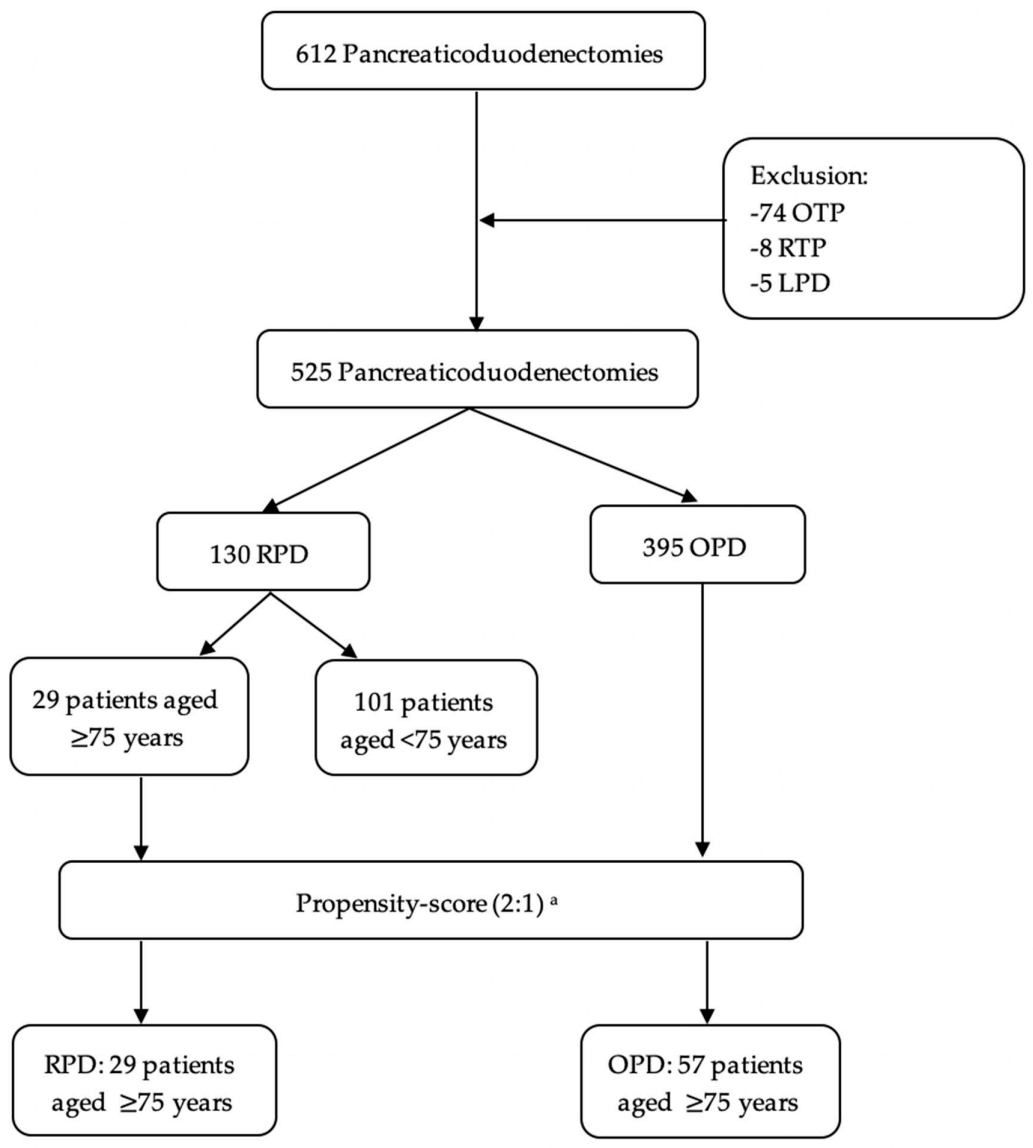
Flow Chart. ^a^ Match according to age, sex, Charlson Comorbidity Index, body mass index, and presumed diagnosis. LPD: Laparoscopic pancreaticoduodenectomy; OPD: Open pancreaticoduodenectomy; OTP: Open total pancreatectomy; RPD: Robotic pancreaticoduodenectomy; RTP: Robotic total pancreatectomy.

Overall, during the study period, 525 patients underwent pancreaticoduodenectomy, of whom 130 (25%) were treated using a robotic approach. Figure S1 illustrates temporal trends in surgical procedures stratified by technique over the period 2019–2025. Within the RPD group, 29 patients (22.3%) were aged ≥ 75 years and were subsequently compared with 57 patients who underwent OPD after 1:2 propensity score matching (Figure 1). The Results section is structured in two parts: first, outcomes of RPD are compared between elderly (≥75 years) and younger patients; second, the safety of RPD in elderly patients is evaluated by comparing RPD with OPD using a propensity score-matched cohort.

### 3.1. RPD Outcomes According to Age Category (≥75 or <75 Years-Old)

Baseline characteristics were largely comparable between elderly and younger patients undergoing RPD, with the exception of ASA score (Table 1).

Overall, postoperative outcomes were similar between age groups. Importantly, age ≥ 75 years was not associated with a higher risk of major postoperative complications or increased 90-day mortality. Rates of pancreatic-specific complications, including clinically relevant POPF, delayed gastric emptying, and postpancreatectomy hemorrhage, were comparable between groups. Notably, the hospital readmission rate was significantly lower in the ≥75-year group (22 (23%) vs. 1 (3.7%), *p* = 0.024) (Table 2).

Univariate and multivariate analyses evaluating factors associated with major postoperative complications are presented in Table 3. In multivariable logistic regression analysis, hypertension was independently associated with an increased risk of major postoperative complications (OR 2.64, 95% CI 1.15–6.11; *p* = 0.022).

### 3.2. Safety of RPD in Elderly Patients Aged ≥ 75 Years: Results of a Propensity Score-Matched Analysis

After propensity score matching, good covariate balance was achieved between the RPD and OPD groups, with standardized mean differences below 0.10 for 18 of the 19 variables included in the matching model. Only one variable (suggested diagnosis of pancreatic adenocarcinoma) remained slightly above the threshold (SMD = 0.104) (Figure S2). Baseline characteristics of the matched cohorts are shown in Table 4. In the matched elderly cohort, the rate of major postoperative complications did not differ between RPD and OPD, and RPD was not associated with an increased risk of major morbidity. Similarly, 90-day mortality was comparable between the two approaches (Table 5). The histopathological results are summarized in Table S1.

With regard to pancreatic-specific complications, RPD was associated with a higher incidence of delayed gastric emptying; however, this difference was mainly driven by mild (grade A) cases. Rates of postoperative pancreatic fistula, clinically relevant POPF, and postpancreatectomy hemorrhage were similar between groups. Notably, RPD was associated with a significantly lower rate of unplanned 30-day readmission compared with OPD (*p* = 0.022).

## 4. Discussion

Age is a complex factor in assessing surgical risk and the benefit–risk balance of PD. Older patients frequently present with a greater burden of comorbidities, increased frailty, and reduced physiological reserve, which collectively expose them to a higher risk of postoperative complications [20,21]. These considerations have traditionally discouraged the use of major surgery in this population. Conversely, the progressive aging of the global population is associated with an increasing incidence of pancreatic disease among elderly individuals, leading to a growing need for surgical management [22,23]. Because of its technical complexity and substantial morbidity and mortality, PD represents a major decision-making challenge in elderly patients.

The results of the present study suggest that patients aged ≥ 75 years do not experience higher rates of major postoperative morbidity or 90-day mortality following RPD, with outcomes comparable to those observed after OPD. In the context of limited access to robotic platforms, these findings support the continued use of RPD in carefully selected patients aged ≥ 75 years, without an associated increase in perioperative risk.

Previous studies have shown that RPD has an acceptable safety and efficacy profile, with potential advantages such as reduced intraoperative blood loss and shorter length of hospital stay, which have contributed to a progressive expansion of its indications [24,25]. However, evidence supporting its use in elderly patients remains limited, particularly in centers with restricted access to robotic systems.

In our experience, outcomes after RPD were first compared between patients aged ≥ 75 years and younger patients. Second, elderly patients undergoing RPD were compared with age-matched patients undergoing OPD using propensity score matching. Consistent with previously published data, no significant differences in postoperative morbidity or mortality were observed between elderly and younger patients undergoing RPD [26]. Notably, a significantly lower readmission rate was observed in patients aged ≥ 75 years. In multivariable analysis, arterial hypertension emerged as an independent risk factor associated with a 2.5-fold increased risk of major postoperative complications. Similarly, as reported by Medeiros et al., age was not identified as an independent risk factor for postoperative morbidity following RPD when elderly patients were compared with those undergoing OPD [9]. These findings remained robust after accounting for the matched design using clustered robust standard errors. Taken together, these findings indicate that chronological age alone is an inadequate surrogate for surgical risk. A more nuanced interpretation should incorporate patient-related factors such as frailty, comorbidity burden, and physiological reserve, which are increasingly recognized as more meaningful determinants of postoperative outcomes in older patients.

The cost associated with robotic platforms is substantial [27]. However, this initial investment may be partially offset by reductions in downstream healthcare expenditures, particularly those related to hospital readmissions and prolonged length of stay, both of which are recognized indicators of surgical care quality [28]. Minimally invasive approaches, including robotic-assisted surgery, are generally associated with faster recovery and reduced postoperative pain. In contrast to the findings reported by Medeiros et al., who observed a higher readmission rate in the robotic group, our study suggests a significant reduction in 30-day readmissions following RPD [9]. A similar trend was observed when comparing RPD outcomes across age groups. However, this finding should be interpreted with caution. The observed difference may partly reflect residual selection bias related to patient allocation, as well as center-specific postoperative care pathways and discharge practices, rather than a true effect of the surgical approach itself. These results should therefore be considered hypothesis-generating and warrant confirmation in larger, multicenter prospective studies. With respect to the length of hospital stay, no significant differences were observed between groups.

Delayed gastric emptying is a common complication following pancreaticoduodenectomy. Although generally considered less severe than postoperative pancreatic fistula, it can be highly debilitating and is associated with prolonged hospitalization when clinically relevant [29]. In the present study, the overall incidence of DGE was higher in the RPD group, in line with the findings of Van Oosten et al. [30]. However, this difference was largely driven by mild (grade A) events, whereas the rates of clinically relevant DGE (grades B and C), which are associated with meaningful clinical impact, were comparable between groups. This distinction is essential to avoid overinterpretation of the observed difference in overall DGE incidence. Available data comparing DGE after RPD and OPD remain heterogeneous. While some studies have reported a higher incidence of DGE after RPD [30], others have suggested a lower rate with the robotic approach [31,32]. This variability likely reflects the influence of technical factors and learning curve effects rather than differences inherent to the surgical approach itself. As previously reported by our group, the incidence of DGE during the implementation phase of RPD appears to be influenced by surgical technique, particularly the configuration of the gastroenterostomy [8]. In that study, modification of the anastomotic site was associated with a progressive decrease in DGE rates, supporting the hypothesis that this complication is at least partly technique-dependent. Although age ≥ 75 years has been described as a potential risk factor for DGE [33], this association was not observed in our cohort when comparing younger and older patients undergoing RPD.

The prolonged operative time observed in this study may be partly explained by the learning curve inherent to this highly complex technique. This learning curve involves not only the console surgeon but also the entire operative team, particularly with respect to patient positioning, docking, and intraoperative workflow. A significant increase in operative time has also been reported in the meta-analysis by Podda et al., supporting the consistency of our findings with the existing literature [34]. Beyond operative duration, the learning curve is also associated with differences in case complexity, as illustrated in our study by the disparity in venous resection rates between groups. This likely reflects a progressive case-selection strategy during the implementation phase of robotic pancreatic surgery, with an initial focus on less complex cases followed by gradual expansion of indications as experience increased, as previously described by Zwart et al. [35]. In this setting, the higher proportion of patients receiving neoadjuvant chemotherapy in the RPD group is also likely attributable to the learning curve. As experience increased, patient selection became progressively less restrictive, with surgeons undertaking more complex cases over time.

This study has several limitations. First, its retrospective design is inherently subject to selection bias, although this was partially mitigated by the use of propensity score matching. In addition, analyses were performed using clustered robust standard errors to account for within-pair correlation and reduce the risk of variance underestimation, thereby strengthening the robustness of the findings. Second, the relatively small number of elderly patients who underwent RPD (n = 29) limits statistical power, particularly for secondary outcomes and multivariable analyses. Therefore, the absence of statistically significant differences in morbidity and mortality should not be interpreted as evidence of equivalence between approaches. Third, most procedures were performed by a single highly experienced surgeon, which may limit the external validity of our findings, especially for centers in the early phase of their robotic learning curve. Fourth, despite the use of propensity score matching, residual confounding cannot be entirely excluded. In particular, a slight imbalance persisted for pancreatic ductal adenocarcinoma, reflecting the structural constraints of implementing robotic pancreatic surgery in a center with limited access to the robotic platform and a progressive expansion of indications during the learning curve. Analyses were limited to a contemporary period in order to minimize time-related bias. Nevertheless, residual confounding related to case selection and disease complexity cannot be excluded and may have influenced treatment allocation. Finally, the single-center design further restricts generalizability. Multicenter studies comparing RPD and OPD in elderly patients are therefore warranted to confirm these findings. Accordingly, the present findings should be interpreted as exploratory and hypothesis-generating rather than as definitive evidence of equivalence between surgical approaches.

## 5. Conclusions

RPD appears to be safe in carefully selected patients aged ≥ 75 years, with no signal of increased perioperative morbidity or mortality compared with younger patients and elderly patients undergoing open surgery. These findings are exploratory and support the selective use of RPD in experienced centers, including settings with limited access to robotic platforms.

## Figures and Tables

**Table 1:** Baseline characteristics of RPD according to age groups.

	Overall (*N* = 130)	<75 Years (*N* = 101)	≥75 Years (*N* = 29)	*p*-Value
Age, years	68 (60–74)	65 (58–70)	78 (76–79)	<0.001
Sex				0.46
Female	75 (58)	60 (59)	15 (52)	
Male	55 (42)	41 (41)	14 (48)	
BMI, kg/m^2^	24.5 (21.1–27.3)	24.4 (20.9–26.8)	25.0 (23.4–27.8)	0.19
Arterial hypertension	41 (32)	28 (28)	13 (45)	0.081
Cardiological history	15 (12)	11 (11)	4 (14)	0.74
Dyslipidemia	20 (15)	12 (12)	8 (28)	0.075
Diabetes	14 (11)	10 (9.9)	4 (14)	0.51
Pulmonary history	20 (15)	17 (17)	3 (10)	0.94
Tobacco use	55 (42)	47 (47)	8 (28)	0.069
Alcohol use	44 (34)	36 (36)	8 (28)	0.42
ASA score				0.038
1	15 (12)	14 (14)	1 (3.4)	
2	104 (80)	76 (75)	28 (97)	
3	11 (8.5)	11 (11)	0 (0)	
Charlson Comorbidity Index				
0	3 (2.3)	3 (3.0)	0 (0)	
1	19 (15)	19 (19)	0 (0)	
2	17 (13)	17 (17)	0 (0)	
3	30 (23)	23 (23)	7 (24)	
4	25 (19)	23 (23)	2 (6.9)	
5	24 (18)	12 (12)	12 (41)	
6	10 (7.7)	3 (3.0)	7 (24)	
8	2 (1.5)	1 (1.0)	1 (3.4)	
Jaundice	48 (37)	38 (38)	10 (34)	0.76
Weight loss	39 (30)	31 (31)	8 (28)	0.75
Presumed diagnosis				0.25
CCA	17 (13)	16 (16)	1 (3.4)	
Duodenal adenocarcinoma	7 (5.4)	6 (5.9)	1 (3.4)	
Lesion of Vater’s ampulla	26 (20)	19 (19)	7 (24)	
Pancreatic ductal adenocarcinoma	44 (34)	35 (35)	9 (31)	
IPMN	23 (18)	15 (15)	8 (28)	
NET	8 (6.2)	5 (5.0)	3 (10)	
Other	5 (3.8)	5 (5.0)	0 (0)	
Preoperative endoscopic drainage	56 (43)	47 (47)	9 (31)	0.14
Preoperative radiologic drainage	1 (0.8)	1 (1.0)	0 (0)	>0.99
Neoadjuvant therapy	25 (19)	23 (23)	2 (6.9)	0.056

ASA: American Society of Anesthesiologists classification; BMI: Body Mass Index; CCA: Cholangiocarcinoma; IPMN: Intraductal papillary mucinous neoplasm; NET: Neuroendocrine tumor; RPD: Robotic pancreaticoduodenectomy.

**Table 2:** Perioperative and postoperative outcomes of RPD according to age groups.

	Overall (*N* = 130)	<75 Years (*N* = 101)	≥75 Years (*N* = 29)	*p*-Value
Operative time, min	575 (550–605)	575 (540–610)	570 (555–580)	0.60
Conversion rate	16 (12)	10 (9.9)	6 (21)	0.19
Pancreatic texture				0.69
Firm	34 (26)	24 (24)	10 (34)	
Intermediate	1 (0.8)	1 (1.0)	0 (0)	
Soft	94 (72.5)	75 (74)	19 (66)	
Intraoperative transfusion	7 (5.4)	5 (5.0)	2 (6.9)	0.65
Venous resection	8 (6.2)	6 (5.9)	2 (6.9)	>0.99
ICU, days	8 (5–13)	7 (5–12)	9 (7–13)	0.16
Length of initial hospital stay, days	14 (9–21)	13 (9–21)	14 (12–21)	0.28
Clavien-Dindo complications				0.41
I/II	96 (74)	73 (72)	23 (79)	
IIIa	15 (12)	14 (14)	1 (3.4)	
IIIb	7 (5.4)	6 (5.9)	1 (3.4)	
IV	5 (3.8)	3 (3.0)	2 (6.9)	
V	7 (5.4)	5 (5.0)	2 (6.9)	
Major complication (Clavien-Dindo ≥ III)	34 (26)	28 (28)	6 (21)	0.45
Reintervention	13 (10)	9 (8.9)	4 (14)	0.48
Delayed gastric emptying	54 (42)	41 (41)	13 (45)	0.68
Grade of delayed gastric emptying				0.14
No	76 (58)	60 (59)	16 (55)	
A	23 (18)	14 (14)	9 (31)	
B	14 (11)	13 (13)	1 (3.4)	
C	17 (13)	14 (14)	3 (10)	
POPF	53 (41)	44 (44)	9 (31)	0.23
CR-POPF	17 (13)	11 (11)	6 (21)	0.21
Grade of POPF				0.091
No	76 (58)	57 (56)	19 (66)	
A	37 (28)	33 (33)	4 (14)	
B	11 (8.5)	8 (7.9)	3 (10)	
C	6 (4.6)	3 (3.0)	3 (10)	
Postoperative hemorrhage	24 (18)	20 (20)	4 (14)	0.46
Chyle leakage	4 (3.1)	2 (2.0)	2 (6.9)	0.22
Sepsis	38 (29)	29 (29)	9 (31)	0.81
Postoperative malnutrition	51 (39)	39 (39)	12 (41)	0.79
Cardiac event	8 (6.2)	3 (3.0)	5 (17)	0.014
Surgical site infection	5 (3.8)	4 (4.0)	1 (3.4)	>0.99
Respiratory event	7 (5.4)	4 (4.0)	3 (10)	0.18
Unplanned hospital readmission (POD 30)	23 (19)	22 (22)	1 (3.6)	0.025
Mortality				
POD 30	7 (5.4)	5 (5.0)	2 (6.9)	0.62
POD 90	10 (7.7)	7 (6.9)	3 (10)	0.43

ICU: Intensive Care Unit; POD: Postoperative day; (CR-)POPF: (Clinically Relevant) Postoperative Pancreatic Fistula; RPD: Robotic pancreaticoduodenectomy.

**Table 3:** Univariate and multivariate analyses of independent factors associated with severe complications (Clavien-Dindo ≥ III) following RPD.

Characteristics	Univariate Analysis			Multivariate Analysis		
	N	OR [95% CI]	*p*-Value	OR	95% CI	*p*-Value
Age	130		0.45			
<75 years		–				
≥75 years		0.68 (0.23–1.76]				
Sex	130		0.804			
Female		–				
Male		1.11 (0.5–2.43)				
BMI		1.03 (0.94–1.13)	0.54			
Arterial hypertension	130		0.026			0.022
No		–				
Yes		2.52 (1.12–5.73)		2.64	(1.15–6.11)	
Dyslipidemia	130		0.67			
No		–				
Yes		1.26 (0.41–3.46)				
Tobacco use	130		0.57			
No		–				
Yes		0.80 (0.35–1.76)				
Alcohol use			0.84			
No		–				
Yes		1.09 (0.47– 2.46)				
ASA score	130	0.83 [0.34–2.0)	0.67			
Jaundice	130		0.29			
No		–				
Yes		0.64 (0.26–1.45)				
Neoadjuvant therapy	130		0.056			0.065
No		–				
Yes		0.33 (0.07–1.03)		0.33	(0.07–1.07)	
Preoperative endoscopic drainage	130		0.59			
No		–				
Yes		1.24 (0.56–2.74)				

ASA: American Society of Anesthesiologists classification; BMI: Body Mass Index; CI: Confidence Interval; OR: Odds Ratio; RPD: Robotic pancreaticoduodenectomy.

**Table 4:** Baseline characteristics of patients aged ≥ 75 years according to the surgical approach used after propensity score matching.

	Overall (*N* = 86)	OPD (*N* = 57)	RPD (*N* = 29)	*p*-Value
Age, years	77.69 (76.27–79.23)	77.70 (76.45–79.69)	77.64 (75.98–79.14)	0.55
Sex				0.70
Female	47 (55)	32 (56)	15 (52)	
Male	39 (45)	25 (44)	14 (48)	
BMI, kg/m^2^	24.2 (22.0–26.9)	23.9 (21.8–26.5)	25.0 (23.4–27.8)	0.083
Tobacco use	26 (30)	18 (32)	8 (28)	0.70
Alcohol use	13 (15)	5 (8.8)	8 (28)	0.029
Arterial hypertension	42 (49)	29 (51)	13 (45)	0.60
Cardiological history	15 (17)	11 (19)	4 (14)	0.52
Dyslipidemia	27 (31)	19 (33)	8 (28)	0.59
Diabetes	13 (15)	9 (16)	4 (14)	>0.99
Pulmonary history	10 (12)	7 (12)	3 (10)	>0.99
CCI				0.29
3	19 (22)	12 (21)	7 (24)	
4	16 (19)	14 (25)	2 (6.9)	
5	33 (38)	21 (37)	12 (41)	
6	16 (19)	9 (16)	7 (24)	
7				
8	2 (2.3)	1 (1.8)	1 (3.4)	
Presumed diagnosis				0.21
Duodenal adenocarcinoma	7 (8.1)	6 (11)	1 (3.4)	
Pancreatic ductal adenocarcinoma	33 (38)	24 (42)	9 (31)	
Lesion of Vater’s ampulla	15 (17)	8 (14)	7 (24)	
CCA	8 (9.3)	7 (12)	1 (3.4)	
Other	2 (2.3)	2 (3.5)	0 (0)	
IPMN	15 (17)	7 (12)	8 (28)	
NET	6 (7.0)	3 (5.3)	3 (10)	
Preoperative endoscopic drainage	28 (33)	19 (33)	9 (31)	0.83
Preoperative radiologic drainage	2 (2.3)	2 (3.5)	0 (0)	0.55
Neoadjuvant therapy	12 (14)	10 (18)	2 (6.9)	0.32

BMI: Body Mass Index; CCA: Cholangiocarcinoma; CCI: Charlson Comorbidity Index; IPMN: Intraductal papillary mucinous neoplasm; NET: Neuroendocrine tumor; OPD: Open pancreaticoduodenectomy; RPD: Robotic pancreaticoduodenectomy.

**Table 5:** Peri- and Postoperative Outcomes of patients aged ≥ 75 years according to surgical approach used (OPD vs. RPD) after propensity score matching.

	Overall (*N* = 86)	OPD (*N* = 57)	RPD (*N* = 29)	*p*-Value
Operative time, min	385 (330–554)	345 (315–385)	570 (555–580)	<0.001
Pancreatic texture				0.77
Firm	32 (38)	22 (39)	10 (34)	
Intermediate	1 (1.2)	1 (1.8)	0 (0)	
Soft	52 (61)	33 (59)	19 (66)	
Intraoperative transfusion	7 (8.1)	5 (8.8)	2 (6.9)	>0.99
Venous resection	17 (20)	15 (26)	2 (6.9)	0.033
ICU, days	9 (7–13)	9 (7–13)	9 (7–13)	0.79
Length of initial hospital stay, days	13 (11–21)	13 (11–21)	14 (12–21)	0.63
Clavien-Dindo complications				0.86
I/II	69 (80)	46 (81)	23 (79)	
IIIa	4 (4.7)	3 (5.3)	1 (3.4)	
IIIb	5 (5.8)	4 (7.0)	1 (3.4)	
IV	4 (4.7)	2 (3.5)	2 (6.9)	
V	4 (4.7)	2 (3.5)	2 (6.9)	
Major complication (Clavien-Dindo ≥ III)	17 (20)	11 (19)	6 (21)	0.88
Reintervention	13 (15)	9 (16)	4 (14)	>0.99
Delayed gastric emptying	24 (28)	11 (19)	13 (45)	0.013
Grade of delayed gastric emptying				0.020
No	62 (72)	46 (81)	16 (55)	
A	14 (16)	5 (8.8)	9 (31)	
B	5 (5.8)	4 (7.0)	1 (3.4)	
C	5 (5.8)	2 (3.5)	3 (10)	
POPF	26 (30)	17 (30)	9 (31)	0.91
CR-POPF	15 (17)	9 (16)	6 (21)	0.57
Grade of POPF				0.95
No	59 (69)	40 (70)	19 (66)	
A	12 (14)	8 (14)	4 (14)	
B	8 (9.3)	5 (8.8)	3 (10)	
C	7 (8.1)	4 (7.0)	3 (10)	
Postoperative hemorrhage	10 (12)	6 (11)	4 (14)	0.73
Chyle leakage	11 (13)	9 (16)	2 (6.9)	0.32
Sepsis	28 (33)	19 (33)	9 (31)	0.83
Postoperative malnutrition	38 (44)	26 (46)	12 (41)	0.71
Cardiac event	6 (7.0)	2 (3.5)	4 (14)	0.17
Surgical site infection	4 (4.7)	3 (5.3)	1 (3.4)	>0.99
Respiratory event	4 (4.7)	1 (1.8)	3 (10)	0.11
Unplanned hospital readmission (POD 30)	13 (16)	12 (21)	1 (3.7)	0.022
Mortality				
POD 30	4 (4.7)	2 (3.5)	2 (6.9)	0.26
POD 90	7 (8.1)	4 (7.0)	3 (10)	0.68

ICU: Intensive Care Unit; POD: Postoperative day; (CR-)POPF: (Clinically Relevant) Postoperative Pancreatic Fistula; OPD: Open pancreaticoduodenectomy; RPD: Robotic pancreaticoduodenectomy.

## Data Availability

Data are available from the corresponding author upon reasonable request.

## Supplementary Materials

The following supporting information can be downloaded at: https://www.mdpi.com/article/10.3390/jcm15041520/s1, Figure S1: Temporal trends in surgical procedures by technique (2019–2025); Figure S2: A Love plot showing covariate balance before and after matching: title; Table S1: Histopathological characteristics of patients aged ≥ 75 years by surgical approach (OPD vs. RPD).

## Abbreviations

ASA	American Society of Anesthesiologists
BMI	Body mass index
CCI	Charlson Comorbidity Index
DGE	Delayed gastric emptying
ICU	Intensive care unit
IPMN	Intraductal papillary mucinous neoplasms
ISGPS	International Study Group of Pancreatic Surgery
OPD	Open pancreaticoduodenectomy
OR	Odds ratio
PD	Pancreaticoduodenectomy
POPF	Postoperative pancreatic fistula
PPH	Postpancreatectomy hemorrhage
RPD	Robotic pancreaticoduodenectomy
SD	Standard deviation
