# Supplementary material for: Interest of the Robotic Approach for Pancreaticoduodenectomy in Elderly Patients in a Setting of Limited Robotic Platform Access: A Propensity Score-Matched Comparison with Open Surgery
Source: J Clin Med. 2026 Feb 14;15(4):1520. doi: 10.3390/jcm15041520 (PMC12942455; doi:10.3390/jcm15041520)
Supplement: Supplementary file 1 [file jcm-15-01520-s001.zip › jcm-4095363-supplementary.pdf]

Supplementary Data

Figure S1: Temporal trends in surgical procedures by technique (2019–2025)

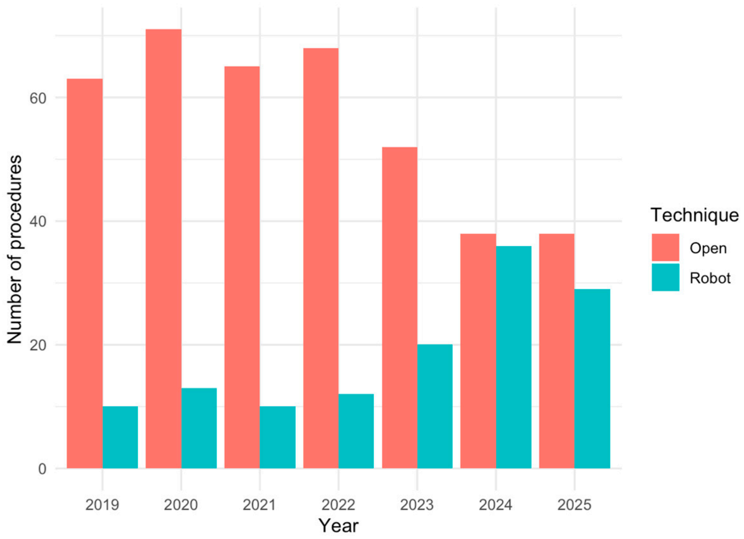

Figure S2: A Love plot showing covariate balance before and after matching

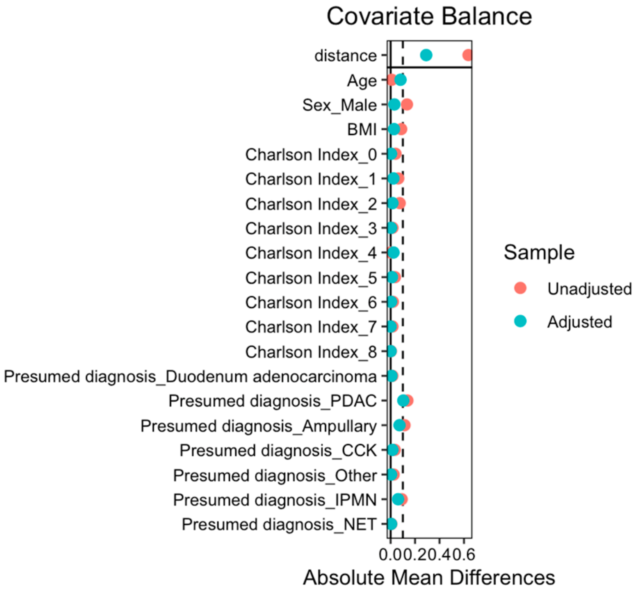

Table S1: Histopathological characteristics of patients aged ≥75 years by surgical approach

	**Overall** (*N = 86*)	**OPD** (*N = 57*)	**RPD** (*N = 29*)	***p*-Value**
Tumor size (mm)	24 (15, 30)	25 (15, 32)	22 (14, 30)	0.3
T stage				0.070
0	11 (14%)	3 (5.9%)	8 (30%)	
is	3 (3.8%)	3 (5.9%)	0 (0%)	
1	22 (28%)	16 (31%)	6 (22%)	
2	27 (35%)	18 (35%)	9 (33%)	
3	13 (17%)	10 (20%)	3 (11%)	
4	2 (2.6%)	1 (2.0%)	1 (3.7%)	
N stage				0.2
0	54 (64%)	32 (57%)	22 (76%)	
1	22 (26%)	18 (32%)	4 (14%)	
2	9 (11%)	6 (11%)	3 (10%)	
M stage				0.5
0	84 (98%)	55 (96%)	29 (100%)	
1	2 (2.3%)	2 (3.5%)	0 (0%)	
R stage				>0.9
R0	80 (93%)	53 (93%)	27 (93%)	
R1	6 (7.0%)	4 (7.0%)	2 (6.9%)	
Lymph nodes harvested	19 (13, 29)	21 (15, 30)	12 (8, 21)	<0.001

Is : in-situ
